# Development and validation of self-reported line drawings for assessment of knee malalignment and foot rotation: a cross-sectional comparative study

**DOI:** 10.1186/1471-2288-10-57

**Published:** 2010-06-18

**Authors:** Sarah L Ingham, Amanda Moody, Abhishek Abhishek, Sally A Doherty, Weiya Zhang, Michael Doherty

**Affiliations:** 1 Academic Rheumatology, Clinical Sciences Building, City Hospital, Nottingham, NG5 1PB, UK

## Abstract

**Background:**

For large scale epidemiological studies clinical assessments and radiographs can be impractical and expensive to apply to more than just a sample of the population examined. The study objectives were to develop and validate two novel instruments for self-reported knee malalignment and foot rotation suitable for use in questionnaire studies of knee pain and osteoarthritis.

**Methods:**

Two sets of line drawings were developed using similar methodology. Each instrument consisted of an explanatory question followed by a set of drawings showing straight alignment, then two each at 7.5° angulation and 15° angulation in the varus/valgus (knee) and inward/outward (foot) directions. Forty one participants undertaking a community study completed the instruments on two occasions. Participants were assessed once by a blinded expert clinical observer with demonstrated excellent reproducibility. Validity was assessed by sensitivity, specificity and likelihood ratio (LR) using the observer as the reference standard. Reliability was assessed using weighted kappa (κ). Knee malalignment was measured on 400 knee radiographs. General linear model was used to assess for the presence of a linear increase in knee alignment angle (measured medially) from self-reported severe varus to mild varus, straight, mild valgus and severe valgus deformity.

**Results:**

Observer reproducibility (κ) was 0.89 and 0.81 for the knee malalignment and foot rotation instruments respectively. Self-reported participant reproducibility was also good for the knee (κ 0.73) and foot (κ 0.87) instruments. Validity was excellent for the knee malalignment instrument, with a sensitivity of 0.74 (95%CI 0.54, 0.93) and specificity of 0.97 (95%CI 0.94, 1.00). Similarly the foot rotation instrument was also found to have high sensitivity (0.92, 95%CI 0.83, 1.01) and specificity (0.96, 95%CI 0.93, 1.00). The knee alignment angle increased progressively from self reported severe varus to mild varus, straight, mild valgus and severe valgus knee malalignment (p_trend _<0.001).

**Conclusions:**

The two novel instruments appear to provide a valid and reliable assessment of self-reported knee malalignment and foot rotation, and may have a practical use in epidemiological studies.

## Background

It is now recognised that biomechanical factors are important in the pathogenesis of osteoarthritis (OA) [1,2]. Angulations of the knees or feet can significantly influence the distribution of stress forces through the lower limb joints. Varus malalignment increases mechanical load transmitted through the medial tibio-femoral compartment during weight-bearing [1], whilst pressures through the lateral tibio-femoral compartment are increased with valgus malalignment [3]. Alteration in foot rotation may also alter the distribution of forces through the tibial plateaux and thus alter stress through the tibio-femoral compartments[4].

The current "gold standard" for determining knee malalignment is weight-bearing full-length leg radiographs [1,5]. The anatomic axis measured from a standard weight-bearing knee radiograph is a more readily undertaken substitute for measuring the mechanical axis compared to a full limb radiograph [6,7]. However, the impracticalities of a weight-bearing full-length leg radiograph and the comparative expense and inconvenience of knee radiographs remains an obstacle for some large scale epidemiological studies and clinical assessment of a standing participant is a practical alternative. No "gold standard" exists for the assessment of foot rotation, though some studies have used digital photography [8] and camera motion analysis [9] to assess this. However, simple line-drawings, suitable for self-reported questionnaires, have been used successfully for self-reporting of bodily pain location [10] and for self-reported assessment of physical characteristics such as hallux valgus [11] and self-reported Heberden's and Bouchard's nodes [12].

The objectives of this study were to develop, and validate against clinical assessment, two instruments for self-reported assessment of knee malalignment and for foot eversion/inversion that may be suitable for epidemiological studies. The self-report knee malalignment instrument was also validated against radiographically assessed knee malalignment.

## Methods

This development and validation formed part of a wider epidemiological study into knee pain [13] that was approved by the local Nottingham1 Research Ethics Committee. All participants provided informed, written consent.

### Development of the self-report instruments

**Figure 1:**
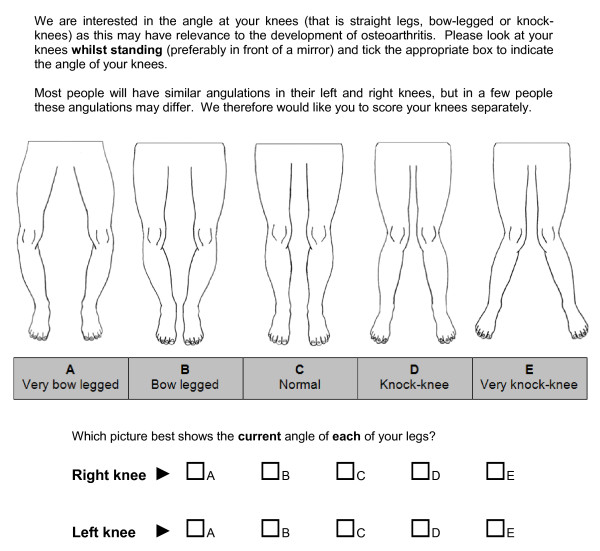
**Novel self-report instrument for assessment of knee malalignment**.

**Figure 2:**
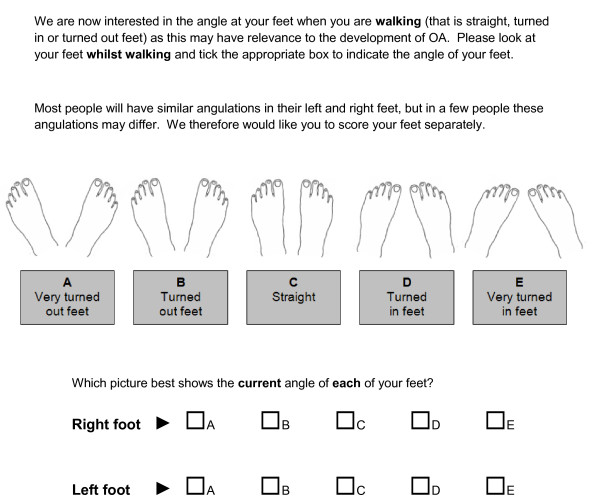
**Novel self-report instrument for assessment of foot rotation**.

Two novel line drawings with various degrees of knee malalignment and foot rotation were created by the Department of Academic Rheumatology using similar methodologies. Initial line drawings of straight aligned knees and feet were drawn (MD) and then adjusted to give a series of drawings depicting interval changes of 7.5° in each direction, that is both varus/outward and valgus/inward directions. Two degrees of severity in either direction were thought to be reasonable to cover the spectrum of clinical abnormality for knee and foot mal-alignment. Both instruments were accompanied by a set of instructions communicating how the drawings should be used to determine current knee (Figure 1) and foot angulation (Figure 2).

### Establishment of reference standard

Observer assessment was used as the reference standard. The reproducibility was undertaken on 10 patients attending the Nottingham Rheumatology Unit out-patient department. These patients were selected because they displayed a range of knee and foot angulations, including some with normal alignment. The same observer classified their angulations as previously described, at two time points on the same day. Reproducibility was high for both the self-reported knee malalignment instrument (κ 0.89; 95%CI 0.59, 1.18) and the foot rotation instrument (κ 0.81; 95%CI 0.42, 1.20).

### Validity and reliability of self reported instruments

Three thousand one hundred and nine participants undertaking a community study into knee pain completed the novel instruments as part of a postal questionnaire, 424 of whom were also seen for a knee pain assessment. Forty one of these clinical participants were randomly selected (using computer-generated random numbers in Excel) to repeat the self-report instruments after approximately 3 weeks, and were blinded to their original response. Participants were asked to look at their knees in a mirror whilst standing. Participants were first shown the instrument for knee malalignment and asked to indicate which picture best represented the current angle of their knees; classifying their left and right knees separately. This method was repeated using the foot rotation instrument except participants were required to look at their feet whilst walking instead of looking in a mirror. A blinded observer then assessed the degree of angulation for both knees and feet using the line drawing questionnaire, blind to the participant derived rating. The validity of the self-reported instruments was determined by the comparison between self-reported measures and the observer measures - the reference standard. The reliability was determined by repeated measures within participants.

### Radiographic validation of self reported knee malalignment instrument

Knee malalignment was measured on weight-bearing postero-anterior knee radiographs in 400 separate participants who self reported their knee alignment using the line diagram instrument. These individuals were part of the Genetics of Osteoarthritis and Lifestyle (GOAL) cohort which has been described previously [14]. The anatomic axis was measured using a published technique [7]. The mechanical axis was estimated by offsetting the anatomic axis by 2° (for women) and 4° (for men) [6]. The estimated mechanical axis was recorded as a continuous variable, the knee alignment angle measured medially with <180° being varus and > 180° being valgus.

### Statistical analysis

Validity was determined by sensitivity, specificity, likelihood ratio (LR) and accuracy. To calculate these outcomes, the data were dichotomised as positive (e.g. varus knee) and negative (e.g. no varus knee) and the observer's assessment was used as a reference standard. Sensitivity is the proportion of true positives that are correctly self-diagnosed by participants, whereas specificity is the proportion of true negative which are correctly classified by participants [15]. The ideal self-reported instrument would have a value of 1 for both sensitivity and specificity that is 100% sensitive and specific. LR summarises how many times more (or less) likely subjects with the condition are to be self-reported as having this condition than those without the condition. An LR greater than 1 indicates the self-reported result is associated with the presence of the condition, whereas LR less than one indicates that the self-reported result is associated with the absence of the condition. LRs above 10 or below 0.1 are considered to be strong evidence to respectively rule in or rule out a diagnosis in most circumstances [16]. Accuracy is a measure of overall agreement between the reference standard (i.e. observer's classification) and the self-reported classification. Standard 2x2 table was formulated to calculate each of these measures and their 95%CIs [15,16].

Reliability was analysed using weighted kappa statistics as there are more than two categories for both the knee and foot instruments and the categories are ordered. 95% confidence interval (CI) of the kappa was calculated using the Fleiss method [17].

All analyses were performed on the left and right body sides separately and the results were combined if necessary to produce an overall result for each outcome, weighted by their sampling variances [18]. StatsDirect version 2.7.3 was used for the analyses.

The most radiographically malaligned knee was selected as the 'index knee' for each individual. General linear model was used to assess for the presence of a linear increase in knee alignment angle from severe varus (group A) to severe valgus (group E) deformity at the knee. Mean (95% CI) knee alignment angle was established for each of the five categories of self-reported knee malalignment. The analysis was carried out using SPSS (v14).

## Results

### Participant demographics

Forty one participants provided data for the self-reported participant reproducibility and for the validity assessment. The age of these participants ranged between 54 and 87 years, with a mean age of 71 years old. Twenty six (63%) of the 41 participants were female. Mean body mass index (BMI) was calculated to be 28.7. The full series of drawings depicting interval clinical changes for both knee malalignment and foot rotation were assessed during the validation.

### Validity of self-reported instruments

The validity of both the knee malalignment and foot rotation instruments is shown in Table 1. Sensitivity, specificity, and accuracy were all close to 100% when assessing varus malalignment using the self-reported knee malalignment instrument. For valgus knee malalignment, calculated values were also extremely high (Table 1). Similarly, the foot rotation instrument appeared to provide a valid assessment of self-reported inward foot rotation (Table 1). For those with feet angled outwards the instrument was also found to be highly sensitive, specific, and accurate (Table 1). Overall instrument validity values and likelihood ratios can also be seen in Table 1.

**Table 1:** Validity data for the self-reporting line drawing instruments

	Sensitivity (95%CI)	Specificity (95%CI)	Likelihood ratio (95%CI)	Accuracy (95%CI)
**Knee**				
Varus	**0.80 (0.45, 1.15)**	**0.98 (0.95, 1.02)**	**45.60 (6.22, 334.11)**	**0.97 (0.92, 1.01)**
Valgus	**0.71 (0.48, 0.95)**	**0.96 (0.92, 1.01)**	**20.00 (4.93, 81.16)**	**0.91 (0.85, 0.98)**
**Overall**	**0.74 (0.54, 0.93)**	**0.97 (0.94, 1.00)**	**21.31 (-15.81, 58.44)**	**0.95 (0.91, 0.99)**
**Foot**				
Inwards	**0.75 (0.33, 1.17)**	**0.98 (0.93, 1.02)**	**33.00 (4.38, 248.40)**	**0.96 (0.90, 1.02)**
Outwards	**0.93 (0.84, 1.02)**	**0.92 (0.84, 0.99)**	**10.97 (4.27, 28.14)**	**0.92 (0.86, 0.98)**
**Overall**	**0.92 (0.83, 1.01)**	**0.96 (0.93, 1.00)**	**11.18 (-0.70, 23.06)**	**0.94 (0.90, 0.98)**

CI - confidence interval

There was a linear trend in increase in knee alignment angle from those with self-reported severe varus to those with mild varus, straight, mild valgus and severe valgus deformity (p < 0.001) (Table 2). On direct comparison, however, there was no difference in knee alignment angle between those with severe and mild self-reported knee malalignment.

**Table 2:** Knee alignment angle for different grades of self reported knee malalignment

Self reported knee malalignment	n	Knee alignment angle Mean (95% CI)
**Severe varus**	15	**172.72 **(169.86, 175.58)
**Mild varus**	29	**172.95 **(171.17, 174.72)
**Straight**	318	**176.76 **(176.14,177.38)
**Mild valgus**	31	**181.61**(179.62,183.60)
**Severe Valgus**	7	**185.79**(181.60, 189.99)
**Overall**	400	**177.97**(176.81,179.12)
		P_trend <0.001_

CI - confidence interval

### Reliability of self-reported instruments

The kappa score for self-reported participant reproducibility was good for the knee malalignment instrument at 0.73 (95%CI 0.56, 0.90). Self-reported participant reproducibility of the foot rotation instrument was found to be excellent, with 87% of participants being able to reproduce exactly the results they reported in their questionnaires (κ 0.87; 95%CI 0.69, 1.06).

## Discussion

This study details the development and validation of two novel, self-reporting instruments for knee malalignment and foot rotation. Validity and reliability scores were high when assessing both of these instruments, supporting their suitability for use in self-reported questionnaires. Therefore, the instruments appear to be sensitive, specific and reliable in the Caucasian population.

To our knowledge, these are the first self-reporting line drawing instruments to be developed for assessment of knee alignment and foot angulation. The instruments are easy to understand and would be ideally suited for use in epidemiological questionnaires, given the low cost and self-reported application. We intentionally selected line drawings over photographic representations. This is because line drawings are more generic and permit the use of the same instrument for both genders and all ages. Furthermore, line drawings make it easier to incorporate a precise interval, rather than ordinal, scale.

Although full length x-rays have been used as a "gold" standard for precise measurement of knee malalignment [1], its clinical application is restricted and the usual routine assessment of knee OA is a standard weight bearing radiograph, ideally in flexion [19]. Assessment of malalignment on this limited knee only view has been shown to correlate well with findings derived from full length x-rays [20]. However, imaging is less accessible and applicable to many clinical or epidemiological studies undertaken in a community or primary care setting. In these situations, clinical assessment may be used more readily. We have therefore developed a self-reported instrument that may be comparable to an expert clinical assessment. For some research questions, and for precise measurement of knee malalignment, further radiographic examination will still be required.

The progressive increase in knee alignment angle from severe varus to severe valgus knee malalignment suggests that this line drawing instrument is a valid ordinal measure of knee malalignment. However, there was no difference in knee alignment angle between those with mild and severe varus deformity at the knee. This may be due to heterogeneity in patient's perception of severity of varus malalignment at the knee. Thus the self-reported knee malalignment instrument is valid in discriminating between varus, straight and valgus knee malalignment but it may not be robust in discriminating between severe and mild varus malalignment. The difference in mean knee alignment angle between severe varus and valgus knee malalignment was just over 13 degrees (Table 2). This suggests that both patients and expert observers over estimate the degree of knee malalignment in comparison to radiographic assessment.

There are several caveats to this study. Firstly, we examined only 5 self-rated options for each instrument (normal plus two options in each direction) and did not investigate whether 3 or 4 severity intervals either side of 'normal' would still have provided reproducible results. Although we considered 2 grades of severity sufficient for a self-reported instrument, it is possible that an instrument with more gradations could still perform well. An additional caveat is that we only used one expert observer to establish the reference. The variation between observers remains unknown. Furthermore, this is the first validation in one single Caucasian population, the results may not be extrapolated to others, therefore validation should always been considered in applying these instrument in different populations.

## Conclusions

In summary, two line drawing instruments for self-reporting of knee malalignment and foot rotation have been developed. Both instruments appear reliable and valid when compared to expert clinical assessment and may prove useful in future large scale epidemiological studies.

## Competing interests

The authors declare that they have no competing interests.

## Authors' contributions

SI contributed to the conception and design of the instruments, conceived and undertook the knee pain study where they were validated, analysed and interpreted the data, and drafted the manuscript. AM made substantial contributions to the acquisition of data for the knee pain study. SD was the clinically trained observer whose results were used as the reference standard for this validation. AA measured knee malalignment, analyzed the data and drafted the manuscript. WZ conceived the knee pain study where they were validated, contributed to the design of the study, participated in the analysis and interpretation of the data, critically revised the manuscript, and gave final approval of the version to be published. MD conceived the knee pain study where the instruments were validated, contributed to the conception and design of the instruments (line drawings completed by MD), critically revised the manuscript, and gave final approval of the version to be published. All authors read and approved the final manuscript.

## Pre-publication history

The pre-publication history for this paper can be accessed here:

http://www.biomedcentral.com/1471-2288/10/57/prepub
